# ﻿Four new species of Mycenasect.Calodontes (Agaricales, Mycenaceae) from northeast China

**DOI:** 10.3897/mycokeys.93.86580

**Published:** 2022-09-27

**Authors:** Zewei Liu, Yupeng Ge, Hui Zeng, Xianhao Cheng, Qin Na

**Affiliations:** 1 Shandong Key Laboratory of Edible Mushroom Technology, School of Agriculture, Ludong University, Yantai 264025, China Ludong University Yantai China; 2 Institute of Edible Mushroom, Fujian Academy of Agricultural Sciences, Fuzhou 350011, China Institute of Edible Mushroom, Fujian Academy of Agricultural Sciences Fuzhou China; 3 National and Local Joint Engineering Research Center for Breeding and Cultivation of Features Edible Mushroom, Fuzhou 350011, China National and Local Joint Engineering Research Center for Breeding and Cultivation of Features Edible Mushroom Fuzhou China

**Keywords:** coniferous forest, new taxa, phylogeny, saprobic, taxonomy

## Abstract

Species of Mycenasect.Calodontes are representative of the *Mycena* genus as a whole and are easily recognised by the pinkish, reddish, purplish to brownish pileus and larger basidiomata. Furthermore, the colour of the pileus in the species of sect. Calodontes often has a transition or changes in different stages and the combination of the colour of the pileus with cystidia and basidiospores can be used to recognise taxa within this section. To date, 19 species of Mycenasect.Calodontes have been reported worldwide. Including our recent description of *M.yuezhuoi*, five species of sect. Calodontes have been recorded in China. During examination of specimens collected in coniferous forests or mixed broadleaf-conifer forests in temperate regions of China, additional taxa assigned to sect. Calodontes were identified. Four new species are recognised, based mostly on characters of the pileus and cystidia. Phylogenetic analysis of sequence data from multiple DNA regions (ITS + *rpb1* + *tef1*) supported the morphological evidence. Here, we propose *M.polycystidiata*, *M.rufobrunnea*, *M.shengshanensis* and *M.subulata* as new species in Mycenasect.Calodontes. Morphological descriptions, line drawings, habitat photos and comparisons with closely-related taxa are provided. A key to the 23 known species of sect. Calodontes is presented.

## ﻿Introduction

Mycenasect.Calodontes (Fr. ex Berk.) Quél. comprises the taxa in *Mycena* (Pers.) Roussel with a pinkish, reddish, purplish to brownish and mostly hygrophanous pileus, interveined lamellae, smooth cheilocystidia and pleurocystidia (if present) and mostly amyloid spores (Fries 1821; Berkeley 1836; Maas Geesteranus 1992a, 1992b; Harder et al. 2010). The group was initially proposed as Agaricustrib.Clitocybe subtrib. *Calodontes* Fries consisting of six species, then elevated to section rank within Agaricussubgen.Clitocybe Fr. ex Berk. and finally assigned to *Mycena* in 1872 (Fries 1821; Berkeley 1836; Quélet 1872). To date, 19 species are known, mostly from Europe and North America, but five species have been described from Asia (specifically, China, India and Peninsular Malaysia) (Smith 1947; Maas Geesteranus 1980; Perry 2002; Grgurinovic 2003; Robich 2003; Chew et al. 2014; Aravindakshan and Manimohan 2015; Aronsen and Læssøe 2016; Na 2019; Liu et al. 2021).

The circumscription of subsections within sect. Calodontes is problematic. Initially, Smith (1947) divided sect. Calodontes into two subsections, *Granulatae* and *Ciliatae*, according to whether the cheilocystidia were smooth or not, but this system was not widely adopted because most of the species have previously been classified in other sections of *Mycena* on account of the red, yellow or orange basidiomata, coloured lamellar edge and echinulate or diverticulate cheilocystidia, pleurocystidia, pileipellis or stipitipellis (Maas Geesteranus 1980, 1992a, 1992b; Perry 2002; Grgurinovic 2003; Robich 2003; Harder et al. 2010; Chew et al. 2014). Based on the lamellar edge colour, spore amyloid reaction and cystidial features, a widely accepted subsectional classification of sect. Calodontes was proposed by Maas Geesteranus (1980) and subsequent taxonomists (Grgurinovic 2003; Harder et al. 2010; Chew et al. 2014). The primary criteria for segregation were dominated by microcharacters: subsect. Purae (Konrad & Maubl.) Maas Geest. with amyloid spores and colourless pleuro- and cheilocystidia; subsect. Violacellae Sing. ex Maas Geest. with inamyloid spores and no pleurocystidia; and subsect. Marginatae J.E. Lange with amyloid spores and pleurocystidia and cheilocystidia with purplish-brown contents (Maas Geesteranus 1980; Grgurinovic 2003; Harder et al. 2010; Chew et al. 2014). Although the infrasectional classification of Maas Geesteranus (1980) is generally accepted, phylogenetic analyses have provided only weak support because subsect. Purae and subsect. Violacellae are polyphyletic (Perry 2002; Grgurinovic 2003; Robich 2003; Harder et al. 2010, 2012, 2013; Chew et al 2014; Na 2019). In studying *Mycenapura* (Pers.) P. Kumm., the type of subsect. Purae, Maas Geesteranus (1992a, 1992b) proposed eight forma based on pileus colour. However, 11 clades have been resolved amongst materials collected from Europe and the Americas, which suggests that there may be additional undescribed taxa in subsect. Purae (Harder et al. 2013).

Including our recent description of *M.yuezhuoi* Z.W. Liu, Y.P. Ge & Q. Na from Kunyushan National Nature Reserve (Yantai, Shandong Province), five species of Mycenasect.Calodontes have been previously recorded in China (Li et al. 2015; Na 2019; Liu et al. 2021). In this paper, we propose an additional four new species classified in sect. Calodontes from the temperate zone of northeast China. The four new species share a unique set of striking morphological characters and contribute to an improved understanding of the classification of sect. Calodontes.

## ﻿Materials and methods

### ﻿Morphological observations

Thirteen fungal specimens were examined in this study, which were mainly collected in coniferous forests and some from mixed broadleaf-conifer forests in 2021. Macrocharacters were recorded from fresh specimens. Colour codes in descriptions follow those of Kornerup and Wanscher (1978). Microcharacters were observed from tissues sampled from dried specimens and rehydrated with 5% potassium hydroxide (KOH) and stained with Congo red (1% [w/v] aqueous solution), if necessary, using a Lab A1 microscope (Carl Zeiss AG, Jena, Germany). The amyloid reaction was tested with Melzer’s Reagent (Clémençon et al. 2004; Horak 2005). Twenty basidiospores were measured per specimen. For the holotype, 40 basidiospores from different basidiomata were selected for measurement. Basidiospore statistics are expressed as (*a*/*b*/*c*) (*d*)*e*-*f*-*g* (*h*) × (*i*)*j*-*k*–*l* (*m*) μm [Q = (*n*)*o*-*p* (*q*), Q = *r* ± *s*], where *a*–*c* represent *a* basidiospores of *b* basidiomata from *c* specimens measured; *d* and *h* are the minimum and maximum length (5% extremum), respectively, *e* and *g* indicate the range of values for the remaining 90% of the spores and *f* is the average length; width (*i*–*m*) and Q values (*n*–*q*) are expressed in a similar manner; and *r* and *s* are the average Q value and its standard deviation, respectively (Ge et al. 2021; Liu et al. 2021; Na et al. 2021; Na et al. 2022). The measurement of basidia, cystidia and other characters were each based on 20 observations. All specimens have been deposited in the Fungarium of the Fujian Academy of Agricultural Sciences (**FFAAS**).

### ﻿DNA extraction, PCR, cloning and DNA sequencing

The Plant Genomic DNA Kit (CoWin Biosciences, Beijing, China) was used to isolate total genomic DNA from dried specimens in accordance with the manufacturer’s instructions. Three nuclear loci were sequenced, comprising the internal transcribed spacer (ITS), RNA polymerase II largest subunit (rpb1) and translation elongation factor-1 alpha (*tef1*). The primer pairs ITS1/ITS4, *rpb1*Mp_f1/*rpb1*Mp_r1 and tEFMp_f2/ tEFMp_r2 were selected to amplify ITS, *rpb1* and *tef1*, respectively (White et al. 1990; Harder et al. 2013; Yu et al. 2020). The PCR reactions were performed in a total volume of 25 µl containing 2 µl DNA template, 1 µl for each primer, 8.5 µl nuclease-free H_2_O and 12.5 µl 2× Utaq PCR MasterMix (ZomanBio, Beijing, China). The PCR protocol for amplification of the ITS region was as follows: 94 °C for 4 min, then 34 cycles of 94 °C for 45 s, 52 °C for 45 s and 72 °C for 1 min, with a final extension of 72 °C for 10 min (Na et al. 2022). The PCR protocol for amplification of the *rpb1* and *tef1* regions followed that of Harder et al. (2013): 94 °C for 60 s, then 10 cycles of 94 °C for 35 s, 53 °C for 45 s and 72 °C for 45 s; then 25 cycles of 94 °C for 35 s, 56 °C for 45 s, 72 °C for 45 s and final extension of 72 °C for 10 min. The PCR products were purified by gel electrophoresis or filter membrane and subjected to Sanger dideoxy sequencing by the Beijing Genomics Institute (Beijing, China).

### ﻿Phylogenetic analysis

A combined ITS, *rpb1* and *tef1* dataset was analysed to infer relationships of the new taxa with other members of sect. Calodontes. We used sequences included in previous studies of sect. Calodontes and from members of the most closely-related section deposited in the GenBank database, which were mainly submitted by Harder et al. (2013), Osmundson et al. (2013), Chew et al. (2014) and Liu et al. (2021). For the analysis, representative species of Mycenasect.Supinae Konrad & Maubl., which is closely related to sect. Calodontes, were selected as the outgroup (Osmundson et al. 2013; Na and Bau 2019). Sequences for each DNA region (ITS, *rpb1* and *tef1*) were aligned in MAFFT version 7 online and the aligned matrices were manually checked with BIOEDIT 7.2.5.0 (Hall 1999; Kuraku et al. 2013; Katoh et al. 2019). The best-fit substitution model for each gene partition was determined with MODELTEST 2.3, based on the Akaike Information Criterion (Posada and Crandall 1998). Maximum Likelihood (ML) analysis was conducted by raxmlGUI 2.09 (Edler et al. 2020). The phylogenetic analysis was performed by a single analysis with six partitions (ITS1, 5.8S, ITS2, *rpb1* exons, *tef1* exons, intron of *rpb1* + introns of *tef1*), using the GTRGAMMA model and 1,000 rapid bootstrap (BS) replicates. For Bayesian Inference (BI), two runs of six chains were run for 15,000,000 generations and sampled every 10,000 generations by MrBayes 3.2.6. At the end of the run, the average deviation of split frequencies was 0.007821, ESS (effective sample size) was 1300.3 and the average Potential Scale Reduction Factor (PSRF) parameter values (excluding NA and > 10.0) = 1.000 and the “sump” and “sumt” commands were used to summarise sampled parameters with 25% burn-in (Ronquist and Huelsenbeck 2003).

## ﻿Results

### ﻿Phylogenetic relationships

**Figure 1.**
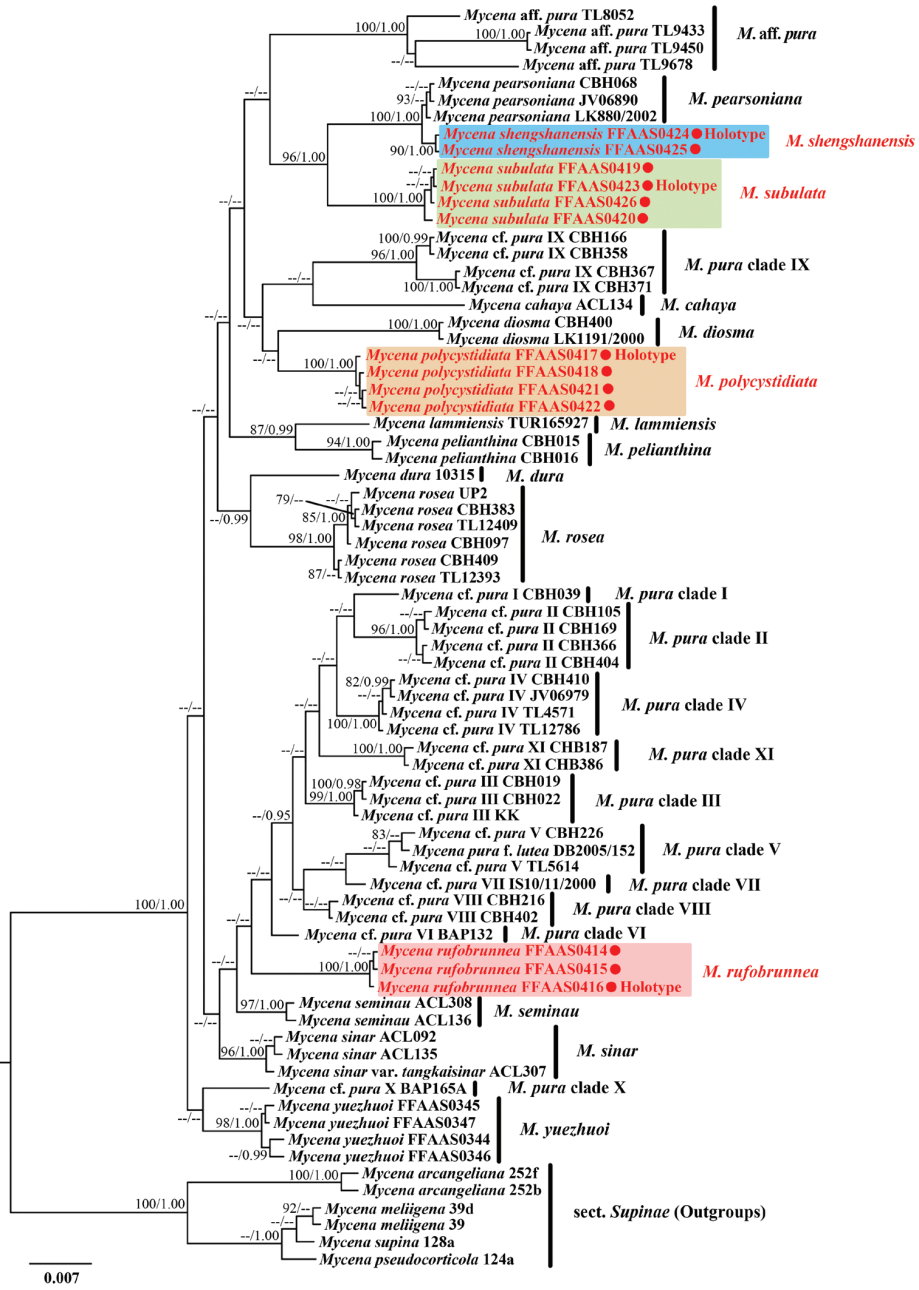
Bayesian Inference analysis of Mycenasect.Calodontes with ITS, *rpb1* and *tef1* sequence data. Species in Mycenasect.Supinae served as outgroup. Bootstrap values (BS) from Maximum Likelihood ≥ 75 and Bayesian posterior probabilities (BPP) ≥ 0.95 are shown on each branch (BS/BPP). The new species are marked in red.

The dataset consisted of 192 sequences, comprising 39 newly-generated sequences (13 ITS, 13 *rpb1* and 13 *tef1*) and 153 sequences (61 ITS, 46 *rpb1* and 46 *tef1*) downloaded from GenBank. In total, 74 accessions of 19 species were included in the dataset. Detailed information for all sequences is presented in Table 1. The aligned dataset contained 1459 nucleotide sites including gaps (229 sites for ITS1, 159 sites for 5.8S, 177 sites for ITS2, 55 sites for *rpb1* exons, 295 sites for *tef1* exons, 544 sites for intron of *rpb1* + introns of *tef1*), of which 1146 were conserved, 257 were parsimony-informative and 56 were variable, but parsimony-uninformative. For Bayesian Inference (BI), the selected models for each DNA region of the concatenated dataset were as follows: HKY+G for ITS1 and intron of *rpb1* + introns of *tef1*, JC for 5.8S and *rpb1* exons, HKY+I+G for ITS2 and SYM+I+G for *tef1* exons. The BI and ML analyses resulted in almost identical topologies; thus, the BI topology is presented as a master tree (Fig. 1).

**Table 1.** Specimens used in phylogenetic analysis and GenBank accession numbers.

No.	Species	Specimen voucher	GenBank accession numbers			Locality	Reference
			ITS	*rpb1*	*tef1*		
1.	Mycenaaff.pura	TL8052	FN394623	KF723687	KF723641	Ecuador	Harder et al. (2010, 2013)
2.	M.aff.pura	TL9433	FN394622	KF723688	KF723642	Ecuador	Harder et al. (2010, 2013)
3.	M.aff.pura	TL9450	KJ144653	KF723689	KF723643	Ecuador	Harder et al. (2010, 2013)
4.	M.aff.pura	TL9678	FN394621	KF723690	KF723644	Ecuador	Harder et al. (2010, 2013)
5.	*M.arcangeliana*	252b	JF908401	–	–	Spain	Osmundson et al. (2013)
6.	*M.arcangeliana*	252f	JF908402	–	–	Spain	Osmundson et al. (2013)
7.	*M.cahaya*	ACL134	KF537248	–	–	Malaysia	Chew et al. (2014)
8.	M.cf.pura I	CBH039	FN394588	KF723680	KF723634	Denmark	Harder et al. (2010, 2013)
9.	M.cf.pura II	CBH105	FN394581	KF723671	KF723625	Denmark	Harder et al. (2010, 2013)
10.	M.cf.pura II	CBH169	FN394579	KF723672	KF723626	Denmark	Harder et al. (2010, 2013)
11.	M.cf.pura II	CBH366	FN394572	KF723673	KF723627	Denmark	Harder et al. (2010, 2013)
12.	M.cf.pura II	CBH404	FN394566	KF723674	KF723628	Denmark	Harder et al. (2010, 2013)
13.	M.cf.pura III	CBH019	FN394605	KF723675	KF723629	Denmark	Harder et al. (2010, 2013)
14.	M.cf.pura III	CBH022	FN394574	KF723676	KF723630	Denmark	Harder et al. (2010, 2013)
15.	M.cf.pura III	KK	FN394606	KF723677	KF723631	Slovakia	Harder et al. (2010, 2013)
16.	M.cf.pura IV	CBH410	FN394595	KF723667	KF723621	Denmark	Harder et al. (2010, 2013)
17.	M.cf.pura IV	JV06979	FN394585	KF723668	KF723622	Denmark	Harder et al. (2010, 2013)
18.	M.cf.pura IV	TL4571	FN394583	KF723669	KF723623	Denmark	Harder et al. (2010, 2013)
19.	M.cf.pura IV	TL12786	FN394591	KF723670	KF723624	Sweden	Harder et al. (2010, 2013)
20.	M.cf.pura V	CBH226	FN394604	KF723664	KF723618	Denmark	Harder et al. (2010, 2013)
21.	M.cf.pura V	TL5614	FN394602	KF723666	KF723620	Denmark	Harder et al. (2010, 2013)
22.	M.cf.pura VI	BAP132	FN394561	KF723660	KF723614	USA	Harder et al. (2010, 2013)
23.	M.cf.pura VIII	CBH216	FN394598	KF723662	KF723616	Denmark	Harder et al. (2010, 2013)
24.	M.cf.pura VIII	CBH402	FN394599	KF723663	KF723617	Denmark	Harder et al. (2010, 2013)
25.	M.cf.pura IX	CBH166	FN394607	KF723701	KF723655	Denmark	Harder et al. (2010, 2013)
26.	M.cf.pura IX	CBH358	FN394608	KF723702	KF723656	Denmark	Harder et al. (2010, 2013)
27.	M.cf.pura IX	CBH367	KF913022	KF723703	KF723657	Denmark	Harder et al. (2013)
28.	M.cf.pura IX	CBH371	KF913023	KF723704	KF723658	Denmark	Harder et al. (2013)
29.	M.cf.pura X	BAP165A	FN394563	KF723698	KF723652	USA	Harder et al. (2010, 2013)
30.	M.cf.pura XI	CBH187	FN394564	KF723678	KF723632	Sweden	Harder et al. (2010, 2013)
31.	M.cf.pura XI	CBH386	FN394565	KF723679	KF723633	Denmark	Harder et al. (2010, 2013)
32.	*M.diosma*	CBH400	FN394617	KF723699	KF723653	Denmark	Harder et al. (2010, 2013)
33.	*M.diosma*	LK1191/2000	FN394619	KF723700	KF723654	Germany	Harder et al. (2010, 2013)
34.	*M.dura*	10315	FN394560	KF723694	KF723648	Austria	Harder et al. (2010, 2013)
35.	*M.lammiensis*	TUR165927	FN394552	KF723697	KF723651	Finland	Harder et al. (2010, 2013)
36.	*M.meliigena*	39	JF908423	–	–	Italy	Osmundson et al. (2013)
37.	*M.meliigena*	39d	JF908429	–	–	Italy	Osmundson et al. (2013)
38.	*M.pearsoniana*	CBH068	FN394614	KF723691	KF723645	Germany	Harder et al. (2010, 2013)
39.	*M.pearsoniana*	JV06890	FN394612	KF723692	KF723646	Denmark	Harder et al. (2010, 2013)
40.	*M.pearsoniana*	LK880/2002	FN394613	KF723693	KF723647	Germany	Harder et al. (2010, 2013)
41.	*M.pelianthina*	CBH015	FN394549	KF723695	KF723649	Denmark	Harder et al. (2010, 2013)
42.	*M.pelianthina*	CBH016	FN394547	KF723696	KF723650	Denmark	Harder et al. (2010, 2013)
**43.**	***M.polycystidiata***	**FFAAS0417 Holotype**	**ON427731**	**ON468456**	**ON468469**	**China**	**This study**
**44.**	***M.polycystidiata***	**FFAAS0418**	**ON427732**	**ON468457**	**ON468470**	**China**	**This study**
**45.**	***M.polycystidiata***	**FFAAS0421**	**ON427733**	**ON468458**	**ON468471**	**China**	**This study**
**46.**	***M.polycystidiata***	**FFAAS0422**	**ON427734**	**ON468459**	**ON468472**	**China**	**This study**
47.	*M.pseudocorticola*	124a	JF908386	–	–	Italy	Osmundson et al. (2013)
48.	*M.pura*	IS10/11/2000	FN394611	–	–	USA	Harder et al. (2010)
49.	M.puraf.lutea	DB2005/152	FN394603	–	–	Denmark	Harder et al. (2010)
50.	*M.rosea*	UP2	FN394550	–	–	UK	Harder et al. (2010)
51.	*M.rosea*	CBH097	FN394556	KF723681	KF723635	Denmark	Harder et al. (2010, 2013)
52.	*M.rosea*	CBH383	FN394553	KF723682	KF723636	Denmark	Harder et al. (2010, 2013)
53.	*M.rosea*	CBH409	FN394551	KF723683	KF723637	Germany	Harder et al. (2010, 2013)
54.	*M.rosea*	TL12393	FN394555	KF723684	KF723638	Denmark	Harder et al. (2010, 2013)
55.	*M.rosea*	TL12409	FN394557	KF723685	KF723639	Denmark	Harder et al. (2010, 2013)
**56.**	***M.rufobrunnea***	**FFAAS0414**	**ON427728**	**ON468453**	**ON468466**	**China**	**This study**
**57.**	***M.rufobrunnea***	**FFAAS0415**	**ON427729**	**ON468454**	**ON468467**	**China**	**This study**
**58.**	***M.rufobrunnea***	**FFAAS0416 Holotype**	**ON427730**	**ON468455**	**ON468468**	**China**	**This study**
59.	*M.seminau*	ACL136	KF537250	–	–	Malaysia	Chew et al. (2014)
60.	*M.seminau*	ACL308	KF537252	–	–	Malaysia	Chew et al. (2014)
**61.**	***M.shengshanensis***	**FFAAS0424 Holotype**	**ON427739**	**ON468464**	**ON468477**	**China**	**This study**
**62.**	***M.shengshanensis***	**FFAAS0425**	**ON427740**	**ON468465**	**ON468478**	**China**	**This study**
63.	*M.sinar*	ACL092	KF537247	–	–	Malaysia	Chew et al. (2014)
64.	*M.sinar*	ACL135	KF537249	–	–	Malaysia	Chew et al. (2014)
65.	M.sinarvar.tangkaisinar	ACL307	KF537251	–	–	Malaysia	Chew et al. (2014)
**66.**	***M.subulata***	**FFAAS0419**	**ON427735**	**ON468460**	**ON468473**	**China**	**This study**
**67.**	***M.subulata***	**FFAAS0420**	**ON427736**	**ON468461**	**ON468474**	**China**	**This study**
**68.**	***M.subulata***	**FFAAS0423 Holotype**	**ON427737**	**ON468462**	**ON468475**	**China**	**This study**
**69.**	***M.subulata***	**FFAAS0426**	**ON427738**	**ON468463**	**ON468476**	**China**	**This study**
70.	*M.supina*	128a	JF908388	–	–	Italy	Osmundson et al. (2013)
71.	*M.yuezhuoi*	FFAAS0344	MW581490	MW868166	MW882249	China	Liu et al. (2021)
72.	*M.yuezhuoi*	FFAAS0345	MW581491	MW868169	MW882250	China	Liu et al. (2021)
73.	*M.yuezhuoi*	FFAAS0346	MW581492	MW868168	MW882251	China	Liu et al. (2021)
74.	*M.yuezhuoi*	FFAAS0347	MW581493	MW868167	MW882252	China	Liu et al. (2021)

The phylogenetic analysis revealed that sect. Calodontes was strong support (BS/Bayesian posterior probability [BPP] = 100/1.00) (Fig. 1). Fifteen species and eleven *M.pura* complex clades within sect. Calodontes were retrieved. Four new species were resolved as monophyletic, each with strong support: *M.polycystidiata* (BS/BPP = 100/1.00), *M.rufobrunnea* (BS/BPP = 100/1.00), *M.shengshanensis* (BS/BPP = 90/1.00) and *M.subulata* (BS/BPP = 100/1.00). A sister relationship between *M.shengshanensis* and *M.pearsoniana* Dennis ex Singer was well supported. *Mycenasubulata* was resolved as sister, but genetically distant from *M.pearsoniana* and *M.shengshanensis* clade. In addition, the sister relationship of *Mycenapolycystidiata* and *M.rufobrunnea* were unresolved.

### ﻿Taxonomy

#### ﻿ Mycena polycystidiata

Taxon classificationFungiAgaricalesMycenaceae

Z.W. Liu, Y.P. Ge, L. Zou & Q. Na, sp. nov.

314F9605-EDAA-5568-B959-48406818050D

MycoBank No: 843977

Figs 2 , 3 , 4 , 5

##### Diagnosis.

Pileus greyish-rose, umbo brownish-orange, hygrophanous. Stipe pubescent. Pleurocystidia polymorphic in shape. Stipitipellis a cutis, with numerous projecting hyphae.

##### Holotype.

China. Heilongjiang Province: Liangshui National Nature Reserve, Yichun City, 47°12'74"N, 128°52'86"E, 20 August 2021, Zewei Liu, Yupeng Ge, Qin Na and Shixin Wang, *FFAAS0417* (collection number MY0633).

##### Etymology.

Refers to the variable shape of pleurocystidia.

##### Description.

***Pileus*** 14–31 mm in diam., campanulate to hemispherical when young, plano-convex with age, with obtuse umbo at centre, margin slightly revolute, at times cracked at mature; umbo brownish-orange (7C3–7C5), disc purplish-grey (13C2, 14B2, 14C2), reddish-grey (12B2, 12C2) to greyish-rose (12B3), near margin reddish-grey (12D2), greyish-ruby (12D3) or purplish-grey (13D3), margin whitish; striate none or indistinct, greyish-ruby (12E3–12E4), towards the centre up to 1/3 diam.; surface dry and rugose, hygrophanous, generally tomentose. ***Context*** white, 2 mm thick, fragile. ***Lamellae*** emarginate, slightly decurrent when old, 20–28 reaching the stipe, 1–2 tiers of lamellulae, white, irregularly intervenose, edge concolorous, wavy. ***Stipe*** 34–73 × 3–7 mm, central, cylindrical, base occasionally compressed with age; apex violet brown (11E3–11E4, 11F4), greyish-ruby (12E4), lower part brownish-grey (11D2, 11E2) to greyish-brown (11D3, 11E3) or purplish-grey (13C2), fragile, hollow; apex to middle densely pubescent, sparser towards base; whitely villose at base. ***Odour*** strongly raphanoid, ***taste*** indistinct.

***Basidiospores*** (130/5/4) (6.4)6.7–7.4–8.3(8.8) × (3.2)3.5–3.9–4.3(4.6) μm [Q = (1.62)1.72–2.05(2.18), Q = 1.90 ± 0.11] [holotype (70/2/1) (6.7)6.9–7.6–8.5(8.7) × (3.4)3.6–4.0–4.4(4.6) μm, Q = (1.71)1.76–2.05(2.13), Q = 1.90 ± 0.09], elongated ellipsoid to cylindrical, colourless, smooth, thin-walled, amyloid. ***Basidia*** 21–31 × 6–8 μm, 4-spored, clavate, hyaline, sterigmata approximately 4 μm in length. ***Cheilocystidia*** thin-walled, hyaline, differs in two shapes, mainly utriform, 50–65 × 20–31 μm, some subclavate, 54–78 × 14–19 μm. ***Pleurocystidia*** abundant, thin-walled, hyaline, multi-shaped: lanceolate and mostly round to blunt apices, 37–81 × 12–20 μm, lanceolate and acute apices, 51–87 × 14–22 μm, elliptical, 30–86 × 12–31 μm, ovate and acute apices, 49–71 × 15–24 μm, ovate and mostly round to blunt apices, 49–73 × 16–22 μm. ***Pileipellis*** a cutis composed of four to five layers cylindrical cells, 51–81 × 4–5 μm, smooth and thin-walled; terminal cells cylindrical or fusiform, 50–69 × 3–22 μm, thin-walled, hyaline. ***Hypodermium*** formed by fusiform to subglobose hyphae, 32–69 × 18–54 μm, thin-walled, hyaline. ***Lamellar trama*** subregular, dextrinoid. ***Stipitipellis*** a cutis composed of cylindrical hyphae 5–8 μm in diam., smooth, thin-walled, with numbers of projecting hyphae 2–6 μm in diam.; ***caulocystidia*** 29–74 × 6–19 μm, clavate or fusiform, thin-walled, smooth, hyaline. ***Clamps*** present in all tissues.

##### Habitat.

Scattered on the litter layers in *Pinuskoraiensis* and *Larixgmelinii* mixed forests.

##### Known distribution.

Heilongjiang Province, China.

##### Additional material examined.

China. Heilongjiang Province: Liangshui National Nature Reserve, Yichun City, 47°12'82"N, 128°52'94"E, 20 August 2021, Zewei Liu, Yupeng Ge, Qin Na and Shixin Wang, *FFAAS0418* (collection number MY0634); same location, 21 August 2021, Zewei Liu, Yupeng Ge, Qin Na and Shixin Wang, *FFAAS0421* (collection number MY0659); same location, 21 August 2021, Zewei Liu, Yupeng Ge, Qin Na and Shixin Wang, *FFAAS0422* (collection number MY0661).

##### Notes.

Macroscopically, *Mycenaluteovariegata* Harder & Læssøe and *M.pura* resemble *M.polycystidiata* in pileus colour, but the latter possesses more typically utriform cheilocystidia and uncontracted pleuro- and cheilocystidia (Perry 2002; Robich 2003; Harder et al. 2013; Aronsen and Læssøe 2016; Na 2019). *Mycenapearsoniana* also has a rose to violaceous pileus, but differs from *M.polycystidiata* in having inamyloid spores and lacking pleurocystidia (Aronsen and Læssøe 2016; Na 2019). Compared with *M.polycystidiata*, *M.sirayuktha* Aravind. & Manim. has similar cheilocystidia, but has an obviously greyish-brown striate pileus, inamyloid spores and slightly glutinous pileipellis with finger-like excrescences (Aravindakshan and Manimohan 2015).

The pleurocystidia of *M.polycystidiata* varied in shape amongst specimens (Fig. 5). In all four specimens, most pleurocystidia were lanceolate and with round to blunt apices, but pleurocystidia with lanceolate and acute apices, elliptical and ovate and acute apices were also observed in *FFAAS0417* (Holotype) and *FFAAS0418*, while elongated lageniform-lanceolate or round apices ovate were detected in *FFAAS0421* and *FFAAS0422*. The multi-shaped pleurocystidia may show a morphological continuum that changes between developmental stages. Nevertheless, the multi-shaped pleurocystidia are unquestionably diagnostic for identification of this species.

**Figure 2.**
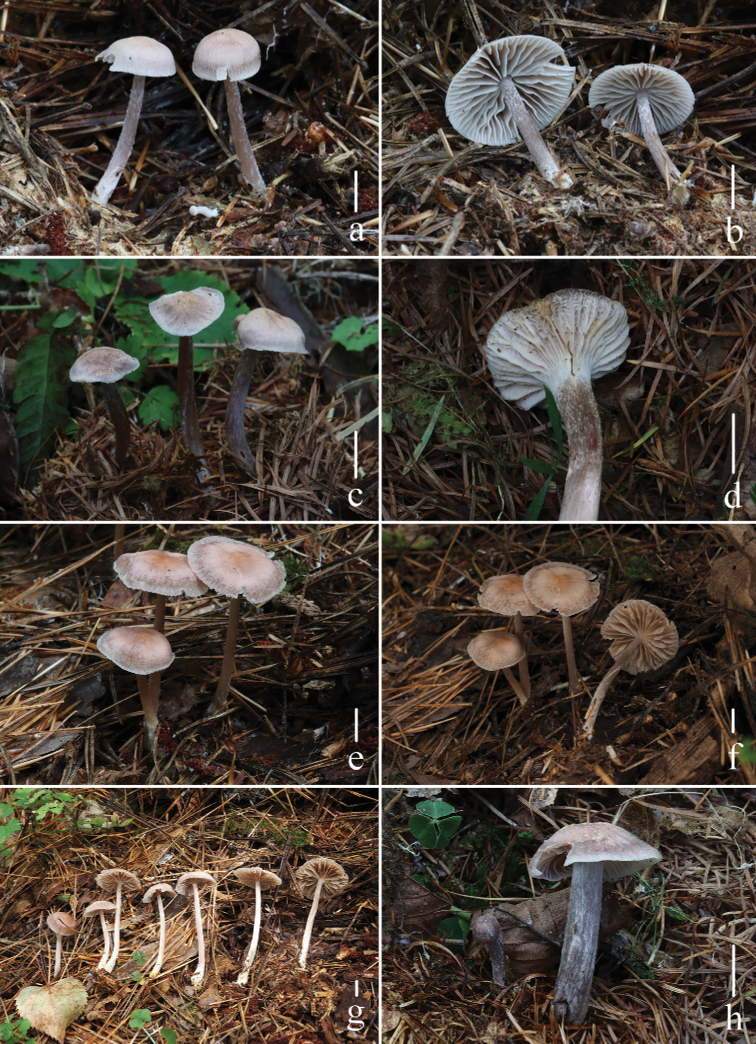
Basidiomata of *Mycenapolycystidiata* Z.W. Liu, Y.P. Ge, L. Zou & Q. Na **a, b***FFAAS0422***c, d***FFAAS0417*, holotype **e–g***FFAAS00421***h***FFAAS0418* Scale bars: 10 mm (**a–h**). Photographs **a–e, h** by Qin Na **f, g** by Yupeng Ge.

**Figure 3.**
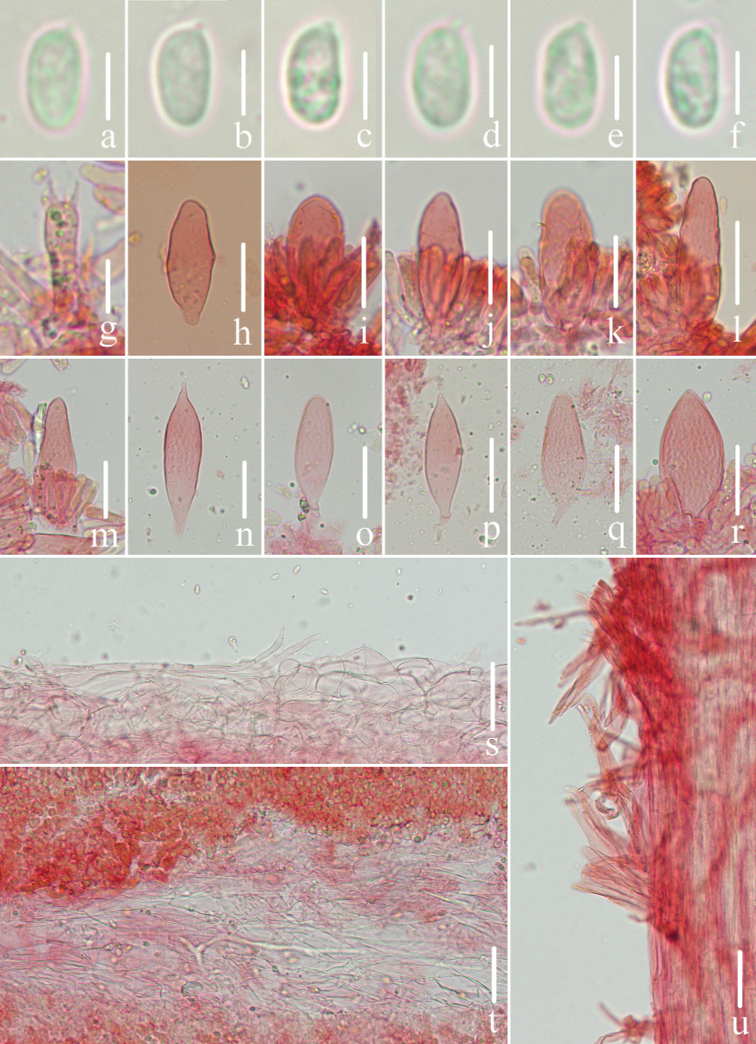
Microscopic features of *Mycenapolycystidiata* (*FFAAS0417*, holotype) **a–f** basidiospores **g** basidia **h–l** cheilocystidia **m–r** pleurocystidia **s** pileipellis and hypodermium **t** lamellar trama **u** stipitipellis and caulocystidia. Scale bars: 5 μm (**a–f**); 10 μm (**g**); 30 μm (**h–r**); 40 μm (**s–u**).

**Figure 4.**
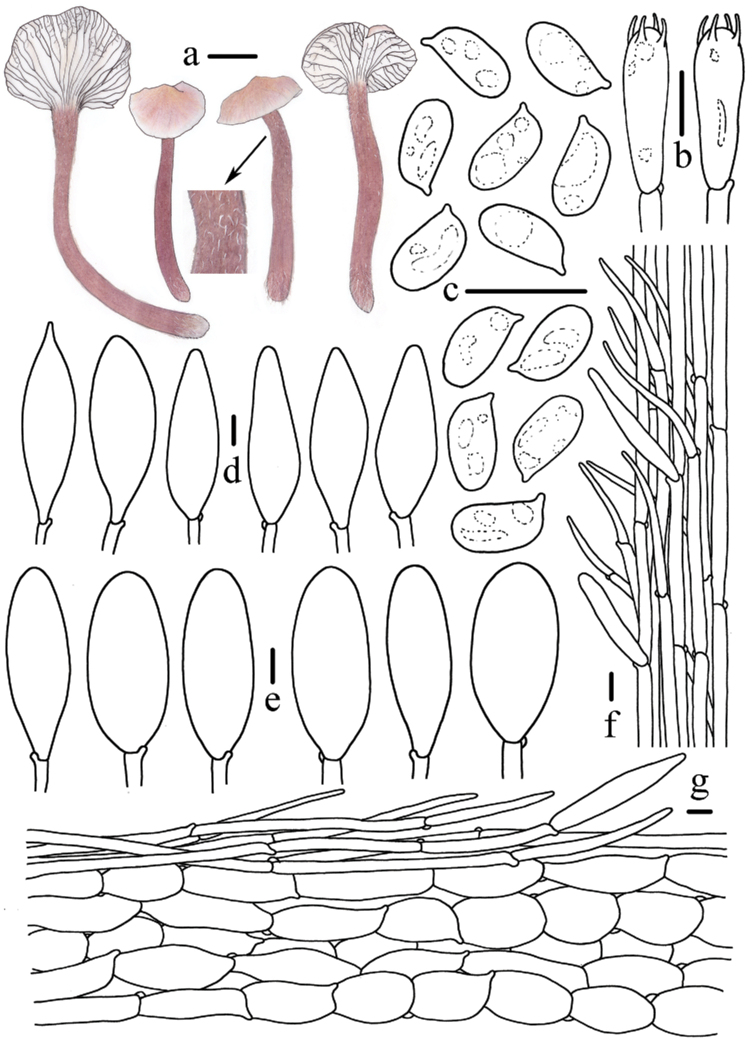
Morphological features of *Mycenapolycystidiata* (*FFAAS0417*, holotype) **a** basidiomata **b** basidia **c** basidiospores **d** pleurocystidia **e** cheilocystidia **f** stipitipellis and caulocystidia **g** pileipellis and hypodermium. Scale bars: 10 mm (**a**); 10 μm (**b–g**). Drawings by Zewei Liu.

**Figure 5.**
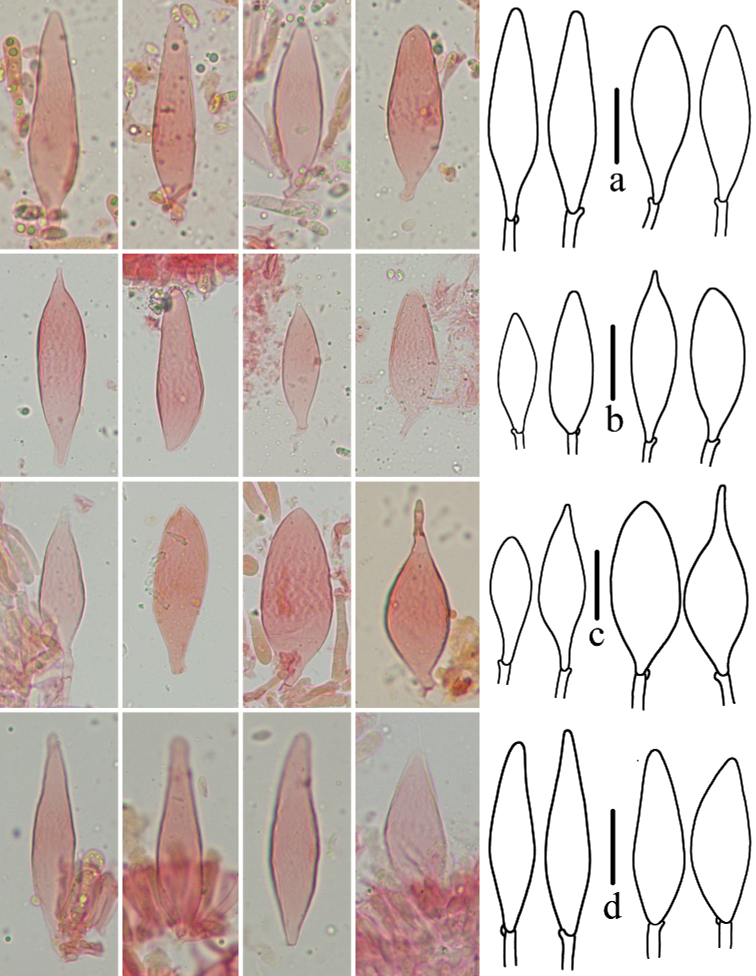
Pleurocystidia of *Mycenapolycystidiata***a***FFAAS0422***b***FFAAS0417*, holotype **c***FFAAS0418***d***FFAAS0421*. Scale bars: 25 μm (**a–d**).

#### ﻿ Mycena rufobrunnea

Taxon classificationFungiAgaricalesMycenaceae

Z.W. Liu, Y.P. Ge & Q. Na, sp. nov.

A5644585-F3C2-536C-B1B0-C0ADB1C6B3C4

MycoBank No: 843978

Figs 6 , 7 , 8

##### Diagnosis.

Pileus dark brown at centre, disc gradually turning paler to reddish-brown to greyish-brown, edge white. Lamellae obviously intervenose. Stipe apex to middle greyish-magenta to dull violet, lower part darker to dark purple or dark magenta. Cheilocystidia utriform, sometimes clavate. Pleurocystidia absent. Caulocystidia clavate or fusiform. Pileipellis with fusiform terminal cells.

##### Holotype.

China. Jilin Province: Dayangcha, Erdaobaihe Town, Antu County, Yanbian Korean Autonomous Prefecture, 42°20'73"N, 127°56'06"E, 16 August 2021, Zewei Liu, Yupeng Ge, Qin Na and Shixin Wang, *FFAAS0416* (collection number MY0581).

##### Etymology.

Refers to reddish-brown pileus.

##### Description.

***Pileus*** 12–34 mm in diam., hemispherical to convex when young, then plano-convex, sometimes an unclear umbo at centre, margin slightly revolute, acute to subacute, at times cracked at mature; dark brown (8F6–8F8) at centre, disc gradually turning paler to reddish-brown (8D4–8D5, 8E6–8E8) to greyish-brown (8D3) and turning to whitish at margin; striate reddish-brown (8D4–8D5, 8E6–8E8), towards the centre up to 1/3–1/2 diam.; surface humidus when wet. ***Context*** white, 1.5 mm thick, fragile. ***Lamellae*** adnexed to emarginate, 20–23 reaching the stipe, 1–3 tiers of lamellulae, white, irregularly intervenose, edge concolorous, slightly serrulate. ***Stipe*** 19–62 × 2–6 mm, central, cylindrical, apex to middle greyish-magenta (14E4–14E5) to dull violet (16E3–16E4), lower part darker to dark purple (14F4–14F5) or dark magenta (13F3), fragile, hollow, base slightly swollen with whitish villose. ***Odour*** raphanoid, ***taste*** indistinct.

***Basidiospores*** (80/4/3) (7.1)7.6–8.4–9.2(9.6) × (3.8)4.0–4.5–5.0 μm [Q = (1.73)1.77–1.98(2.05), Q = 1.88 ± 0.07] [holotype (40/2/1) (7.9)8.1–8.6–9.2(9.4) × 4.2–4.6–5.0 μm, Q = (1.73)1.77–1.96(1.98), Q = 1.87 ± 0.06], elongated ellipsoid to cylindrical, colourless, smooth, thin-walled, amyloid. ***Basidia*** 24–34 × 7–10 μm, 4-spored, clavate, hyaline, sterigmata 2–3 μm in length. ***Cheilocystidia*** thin-walled, hyaline, utriform, sometimes clavate, 23–44 × 7–17 μm, abundant. ***Pleurocystidia*** absent. ***Pileipellis*** a cutis composed of four to five slightly interwoven layers of cylindrical cells, 44–70 × 4–7 μm, smooth, thin-walled; terminal cells cylindrical or fusiform, 34–65 × 4–17 μm, thin-walled, hyaline. ***Hypodermium*** formed by fusiform, subcylindrical to subglobose hyphae, 15–50 × 12–37 μm, thin-walled, hyaline. ***Lamellar trama*** subregular, dextrinoid. ***Stipitipellis*** a cutis composed of hyphae 3–9 μm in diam., smooth, thin-walled; ***caulocystidia*** common in the apex, sparse in the middle and base, 23–76 × 6–14 μm, clavate and fusiform, thin-walled, hyaline, smooth. ***Clamps*** present in all tissues.

##### Habitat.

Scattered on the decayed logs of *Acer*, *Larix*, *Pinus*, *Populus*, *Quercus* and *Ulmus* mixed forests.

##### Known distribution.

Jilin Province, China.

##### Additional material examined.

China. Jilin Province: Dayangcha, Erdaobaihe Town, Antu County, Yanbian Korean Autonomous Prefecture, 42°20'72"N, 127°56'08"E, 16 August 2021, Zewei Liu, Yupeng Ge, Qin Na and Shixin Wang, *FFAAS0414* (collection number MY0579); same location, 16 August 2021, Zewei Liu, Yupeng Ge, Qin Na and Shixin Wang, *FFAAS0415* (collection number MY0580).

##### Notes.

Species of sect. Calodontes that are macroscopically similar to *Mycenarufobrunnea* have been recorded in many regions of the world. Most taxa resemble *M.rufobrunnea* in pileus colour (Smith 1947; Maas Geesteranus 1992a, 1992b; Grgurinovic 2003; Robich 2003; Chew et al. 2014; Aronsen and Læssøe 2016). *Mycenadura* Maas Geest. & Hauskn., recorded in Europe, also has a dark brown to greyish-brown pileus, but can be distinguished from *M.rufobrunnea* in having a white stipe and having pleurocystidia (Robich 2003; Aronsen and Læssøe 2016). *Mycenakuehneriana* A.H. Sm., which is recorded from the United States and Canada, is distinguished from *M.rufobrunnea* in that its pileus is pale avellaneous with rose and lilac, almost white when faded and the spores are obviously smaller (5–6 × 2–3 μm) (Smith 1947; Maas Geesteranus 1992a, 1992b). *Mycenaclarkeana* Grgur. and *M.nullawarrensis* Grgur., described from Australia, are similar to *M.rufobrunnea* in having a reddish-brown pileus, but both species have broader spores and possess pleurocystidia (Grgurinovic 2003). *Mycenacahaya* A.L.C. Chew & Desjardin, *M.seminau* A.L.C. Chew & Desjardin and *M.sinar* A.L.C. Chew & Desjardin, known from Malaysia, resemble *M.rufobrunnea* owing to the brown pileus, but differ in having adnate to subdecurrent lamellae, a yellowish-grey or brownish-orange stem, mucronate cheilocystidia and lack caulocystidia (Chew et al. 2014). Microscopically, utriform or clavate cheilocystidia and absence of pleurocystidia are key characteristics of *M.rufobrunnea*. *Mycenadiosma* Krieglst. & Schwöbel has similar cheilocystidia and pleurocystidia are absent or rare, but it has a strongly hygrophanous pileus and a remarkable change in colour (Robich 2003; Aronsen and Læssøe 2016). *Mycenapura*, *M.sirayuktha* and *M.vinacea* Cleland have similar cheilocystidia, but are easily distinguished from *M.rufobrunnea* by the presence of pleurocystidia (Perry 2002; Grgurinovic 2003; Robich 2003; Aravindakshan and Manimohan 2015; Aronsen and Læssøe 2016; Na 2019).

**Figure 6.**
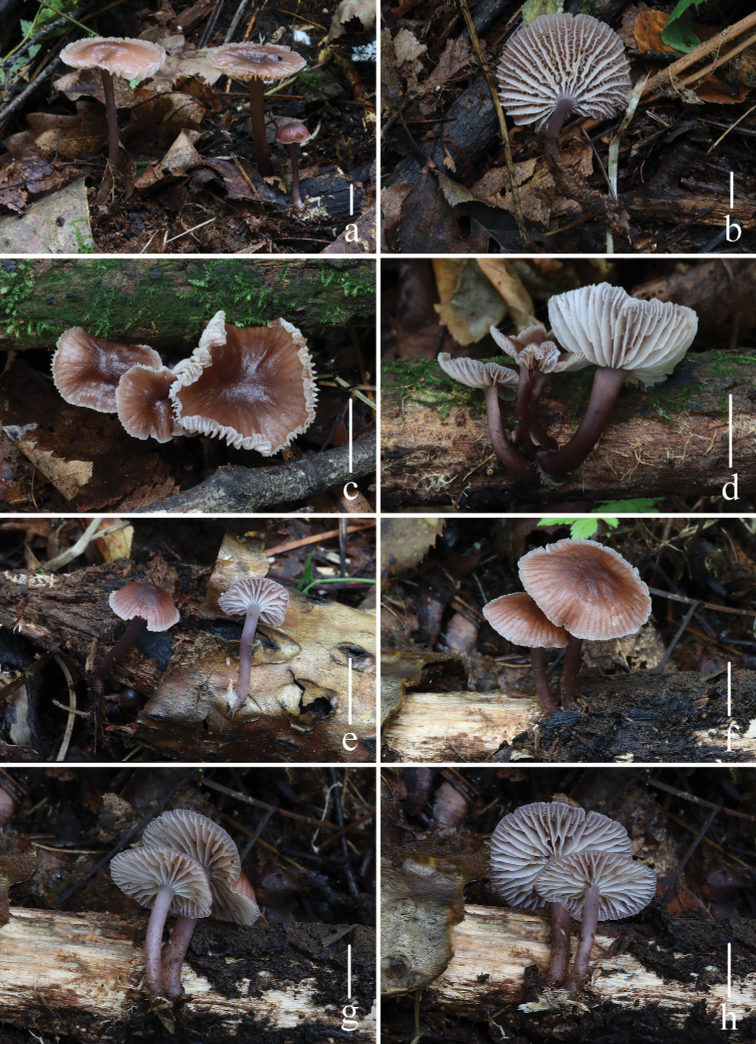
Basidiomata of *Mycenarufobrunnea* Z.W. Liu, Y.P. Ge & Q. Na **a, b***FFAAS0414***c, d***FFAAS0415***e–h***FFAAS0416*, holotype. Scale bars: 10 mm (**a–h**). Photographs **a–h** by Qin Na.

**Figure 7.**
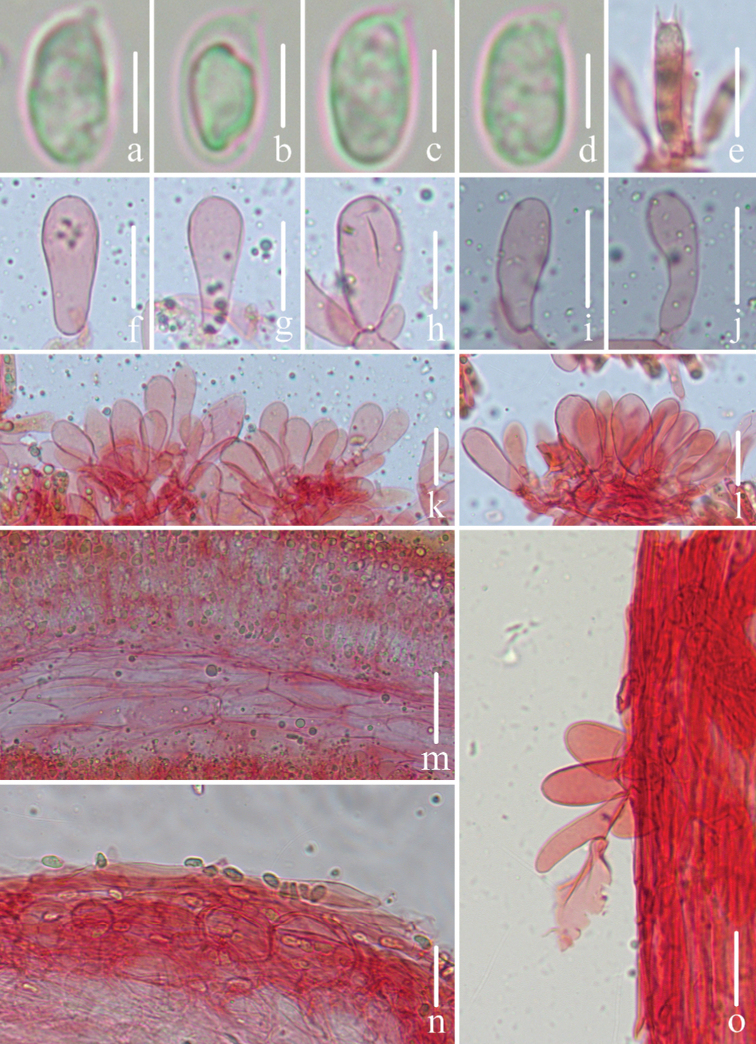
Microscopic features of *Mycenarufobrunnea* (*FFAAS0416*, holotype) **a–d** basidiospores **e** basidia **f–l** cheilocystidia **m** lamellar trama **n** pileipellis and *hypodermium***o** stipitipellis and caulocystidia. Scale bars: 5 μm (**a–d**); 20 μm (**e–o**).

**Figure 8.**
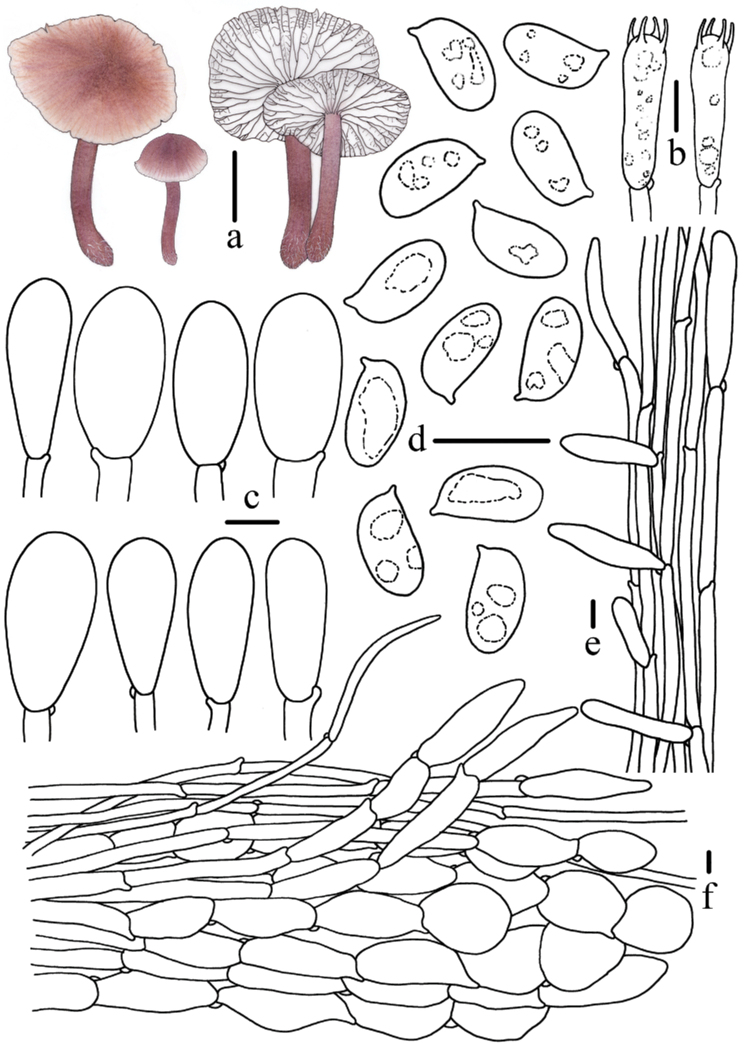
Morphological features of *Mycenarufobrunnea* (*FFAAS0416*, holotype) **a** basidiomata **b** basidia **c** cheilocystidia **d** basidiospores **e** stipitipellis and caulocystidia **f** pileipellis and hypodermium. Scale bars: 10 mm (**a**); 10 μm (**b–f**). Drawings by Zewei Liu.

#### ﻿ Mycena shengshanensis

Taxon classificationFungiAgaricalesMycenaceae

Z.W. Liu, Y.P. Ge & Q. Na, sp. nov.

53D69B3C-50BD-5DF4-8FBD-BF102F7EE427

MycoBank No: 843979

**Figure 9.**
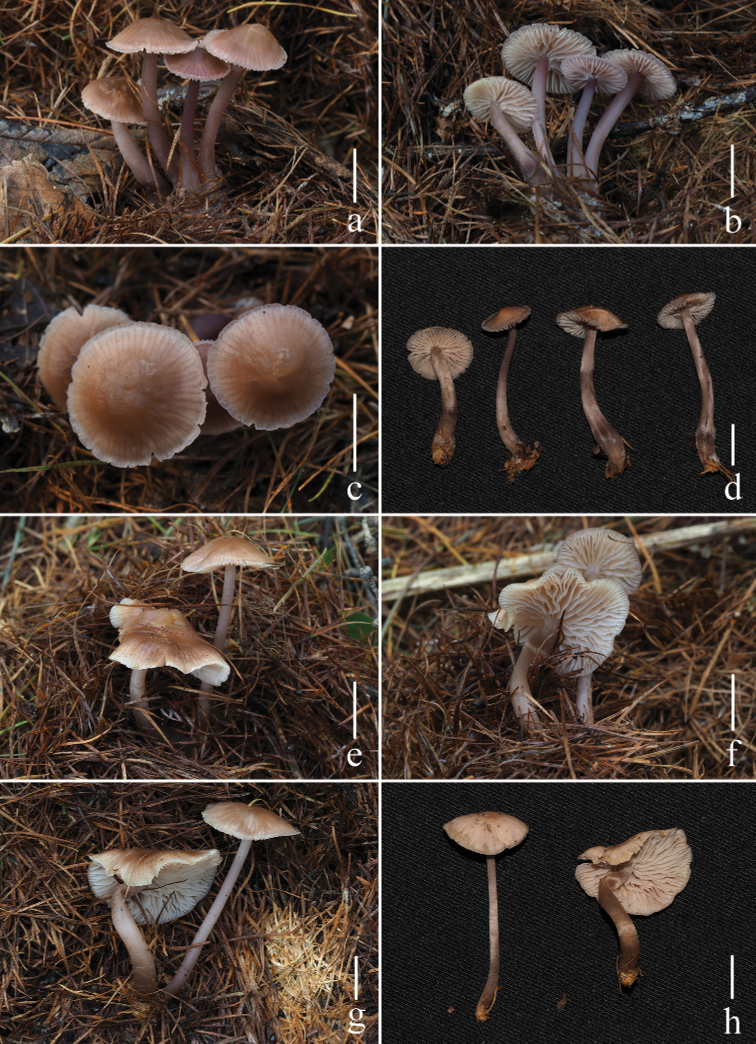
Basidiomata of *Mycenashengshanensis* Z.W. Liu, Y.P. Ge & Q. Na **a–d***FFAAS0424*, holotype **e–h***FFAAS0425* Scale bars: 10 mm (**a–h**). Photographs **a–c, e–g** by Yupeng Ge **d, h** by Zewei Liu.

**Figure 10.**
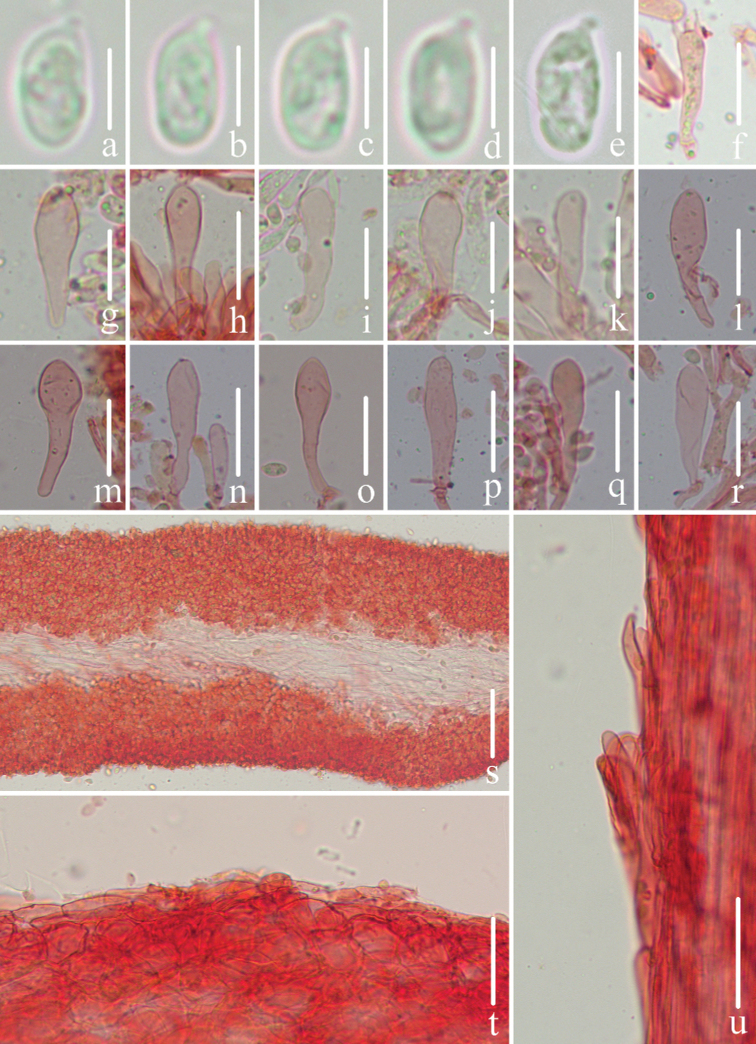
Microscopic features of *Mycenashengshanensis* (*FFAAS0424*, holotype) **a–e** basidiospores **f** basidia **g–r** cheilocystidia **s** lamellar trama **t** pileipellis and hypodermium **u** stipitipellis and caulocystidia. Scale bars: 5 μm (**a–e**); 20 μm (**g–r**); 40 μm (**s–u**).

**Figure 11.**
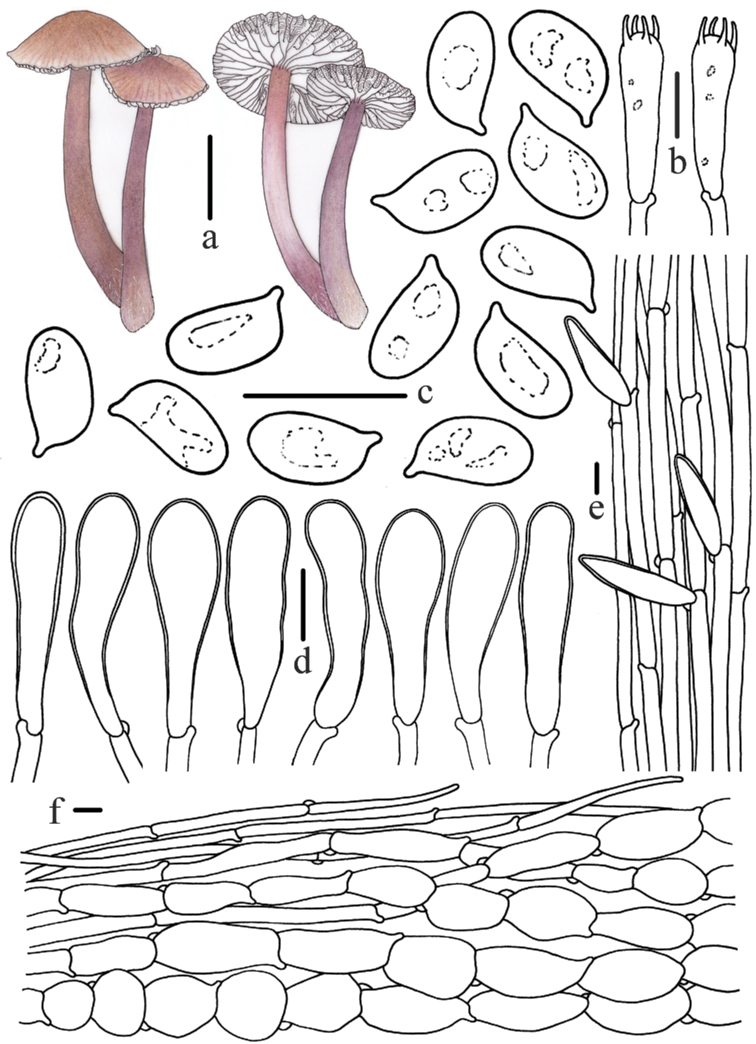
Morphological features of *Mycenashengshanensis* (*FFAAS0424*, holotype) **a** basidiomata **b** basidia **c** basidiospores **d** cheilocystidia **e** stipitipellis and caulocystidia **f** pileipellis and hypodermium. Scale bars: 10 mm (**a**); 10 μm (**b–f**). Drawings by Zewei Liu.

**Figure 12.**
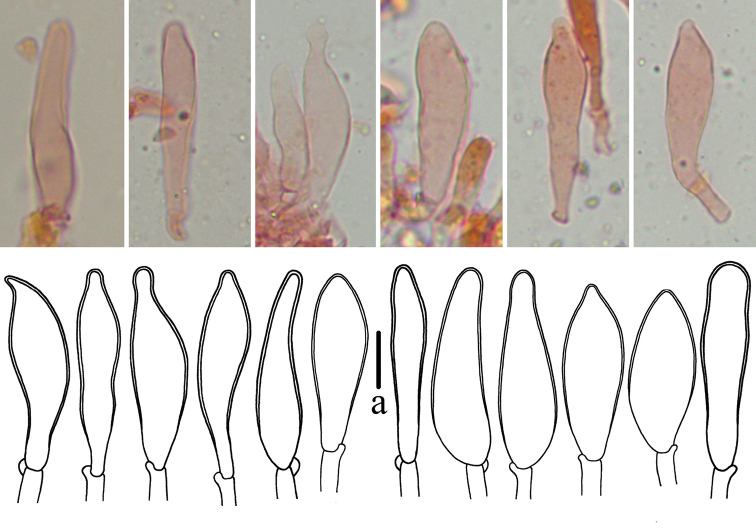
Cheilocystidia of *Mycenashengshanensis FFAAS0424*, holotype. Scale bars: 10 μm (**a**).

Figs 9 , 10 , 11 , 12

##### Diagnosis.

Pileus brown to violet-brown at centre, disc light brown to dull red. Cheilocystidia clavate with slightly inflated apex, thick-walled. Pleurocystidia absent. Caulocystidia clavate with tapered apices, apex to middle thick-walled. Scattered to gregarious under *Larixgmelinii*.

##### Holotype.

China. Heilongjiang Province: Shengshan National Nature Reserve, Heihe City, 49°37'45"N, 126°47'39"E, 23 August 2021, Zewei Liu, Yupeng Ge, Qin Na and Shixin Wang, *FFAAS0424* (collection number MY0686).

##### Etymology.

Refers to the type locality.

##### Description.

***Pileus*** 13–26 mm in diam., when young parabolic to convex, with obtuse umbo at centre, then plano-convex, margin wavy and revolute, at times cracked at mature; centre light brown (7D5–7D6), brown (7E4–7E8), dark brown (8F5–8F6), violet brown (11F4–11F6), disc paler to light brown (7D4–7D5), brown (7E5–7E6), greyish-brown (8D3), reddish-brown (8D4, 8E4), brownish-grey (11C2), dull red (11C3), margin whitish; striate indistinct, brownish-orange (7C3), greyish-brown (7D3), reddish-grey (12D2), greyish-ruby (12E3–12E5), towards the centre up to 1/3–1/2 diam.; surface slightly moist, smooth. ***Context*** white, 1–2 mm thick, fragile. ***Lamellae*** sinuate to subdecurrent, 19–25 reaching the stipe, 1–3 tiers of lamellulae, white, irregularly intervenose, edge concolorous, wavy and slightly serrulate. ***Stipe*** 26–42 × 2–4 mm, central, cylindrical, apex reddish-brown (8E4–8E5), greyish-ruby (12E4–12E5), greyish-brown (11F3), violet brown (11F4–11F5), dark ruby (12F4–12F5), lower part paler to brownish-grey (7C2), brownish-orange (7C3), greyish-brown (11E3), greyish-ruby (12E3), base darker to brown (7E5), reddish-brown (8E4–8E5), greyish-brown (11F3), violet brown (11F4–11F5), greyish-ruby (12E3), dark ruby (12F6–12F8), fragile, hollow, base swollen with white fibrils. ***Odour*** raphanoid, ***taste*** indistinct.

***Basidiospores*** (60/3/2) (5.9)6.1–6.9–8.1(8.7) × 3.4–4.0–4.4(4.7) μm [Q = 1.62–1.93 (1.97), Q = 1.75 ± 0.09] [holotype (40/2/1) 6.1–7.0–8.2(8.7) × 3.4–4.0–4.4(4.7) μm, Q = (1.62)1.65–1.93(1.97), Q = 1.77 ± 0.08], elongated ellipsoid, colourless, smooth, thin-walled, amyloid. ***Basidia*** 22–32 × 6–8 μm, 4-spored, clavate, hyaline, sterigmata 3–4 μm in length. ***Cheilocystidia*** moderately thick-walled (0.5–0.6 μm), clavate with slightly inflated apex, 25–63 × 6–12 μm, hyaline. ***Pleurocystidia*** absent. ***Pileipellis*** a cutis composed of three to four layers of cylindrical cells, 24–57 × 3–5 μm, smooth and thin-walled; terminal cells cylindrical, apically narrow, 28–49 μm in length, apex 1–3 μm and base 2–5 μm in diam., thin-walled, hyaline. ***Hypodermium*** formed by fusiform to subglobose hyphae, 19–53 × 13–30 μm, thin-walled, hyaline. ***Lamellar trama*** subregular, weakly dextrinoid to dextrinoid. ***Stipitipellis*** a cutis composed of hyphae 4–9 μm in diam., smooth, thin-walled; ***caulocystidia*** 22–61 × 5–20 μm, clavate with tapered apices, apex to middle thick-walled, smooth, hyaline. ***Clamps*** present in all tissues.

##### Habitat.

Scattered to gregarious on the litter layer in *Larixgmelinii*.

##### Known distribution.

Heilongjiang Province, China.

##### Additional material examined.

China. Heilongjiang Province: Shengshan National Nature Reserve, Heihe City, 23 August 2021, Zewei Liu, Yupeng Ge, Qin Na and Shixin Wang, *FFAAS0425* (collection number MY0687).

##### Notes.

In sect. Calodontes, *M.diosma*, *M.pearsoniana* and *M.yuezhuoi* also have clavate cheilocystidia with a slightly inflated apex and lack pleurocystidia, similar to *M.shengshanensis*, but *M.diosma* differs in pileus characters, *M.pearsoniana* has inamyloid spores and *M.yuezhuoi* has a more purple pileus and subcellular lamellar trama (Robich 2003; Aronsen and Læssøe 2016; Na 2019; Liu et al. 2021). Clavate cheilocystidia are also present in *M.luteovariegata* and *M.pura*, but these species differ in having pleurocystidia (Perry 2002; Robich 2003; Harder et al. 2013; Aronsen and Læssøe 2016; Na 2019). Macroscopically, several species in sect. Calodontes also have a brown with reddish or violet pileus or stipe (Smith 1947; Maas Geesteranus 1992a, 1992b; Grgurinovic 2003; Robich 2003; Aronsen and Læssøe 2016). *Mycenadura*, *M.kuehneriana* and *M.nullawarrensis* are distinguished by basidiospore size (Smith 1947; Maas Geesteranus 1992a, 1992b; Grgurinovic 2003; Robich 2003; Aronsen and Læssøe 2016). Two species, *M.seminau* and *M.sirayuktha*, reported from Southeast Asia, are similar to *M.shengshanensis* owing to the brown pileus, but they differ in having gelatinised or sometimes mucronate cheilocystidia and caulocystidia have not been observed (Chew et al. 2014; Aravindakshan and Manimohan 2015). Fusiform, obclavate, ovate and clavate cheilocystidia with a subcapitate protuberance were observed occasionally, but clavate cheilocystidia with a slightly inflated apex represented the predominant morphological type in *M.shengshanensis* (Fig. 12).

#### ﻿ Mycena subulata

Taxon classificationFungiAgaricalesMycenaceae

Z.W. Liu, Y.P. Ge & Q. Na, sp. nov.

E08A7A12-E21E-5CD0-B4D9-2B1C693B32FC

MycoBank No: 843980

Figs 13 , 14 , 15

##### Diagnosis.

Pileus reddish-grey to dull red, slightly hygrophanous. Cheilocystidia thick-walled, slenderly fusiform with distinctly long and narrow protuberance. Stipitipellis a cutis, with projecting hyphae, caulocystidia thick-walled.

##### Holotype.

China. Heilongjiang Province: Liangshui National Nature Reserve, Yichun City, 47°13'13"N, 128°53'21"E, 21 August 2021, Zewei Liu, Yupeng Ge, Qin Na and Shixin Wang, *FFAAS0423* (collection number MY0671).

##### Etymology.

Refers to cheilocystidia with distinctly long and narrow protuberance.

##### Description.

***Pileus*** 9–32 mm in diam., convex to campanulate when young, hemispherical to applanate with age, margin sometimes wavy, slightly deflexed; at centre dull red (8C3), brownish-grey (8D2), greyish-brown (8D3), reddish-brown (8D4, 8E4–8E5) and dark brown (8F5), disc paler to reddish-grey (8B2, 9B2), brownish-grey (9C2), dull red (9B3, 9C3), greyish-magenta (13D3), margin light brown (7D4), brown (7E4) or dull red (9C3); striate none or indistinct, reddish-brown (8E4–8E5), towards the centre up to 1/5 diam.; surface dry, unclearly rugose or none, margin slightly hygrophanous. ***Context*** white, 1 mm thick, fragile. ***Lamellae*** sinuate to subdecurrent, 31–33 reaching the stipe, 1–3 tiers of lamellulae, white, irregularly intervenose, edge concolorous, wavy and slightly serrulate. ***Stipe*** 27–75 × 2–5 mm, central, cylindrical, apex to middle brownish-orange (7C3), dull red (8C3), brownish-grey (7D2), greyish-magenta (14D3), lower part brownish-grey (8C2), greyish-brown (8D3), reddish-brown (8D4), fragile, hollow, white granular near apex, base slightly swollen with white fibrils. ***Odour*** raphanoid, ***taste*** indistinct.

***Basidiospores*** (100/5/4) 6.0–6.7–7.3(7.9) × 3.3–3.8–4.3(4.6) μm [Q = (1.61)1.65–1.87(1.90), Q = 1.76 ± 0.07] [holotype (40/2/1) (6.0)6.2–6.6–7.1 × 3.4–3.7–4.0(4.2) μm, Q = 1.65–1.87(1.90), Q = 1.78 ± 0.07], elongated ellipsoid, colourless, smooth, thin-walled, amyloid. ***Basidia*** 23–34 × 5–6 μm, 4-spored, clavate, hyaline, sterigmata 2–3 μm in length. ***Cheilocystidia*** moderately thick-walled (0.5–0.6 μm), hyaline, narrowly fusiform with long and narrow protuberance, 43–82 × 4–11 μm, protuberance 14–36 × 1–2 μm. ***Pleurocystidia*** absent. ***Pileipellis*** a cutis composed of three to four layers of cylindrical cells, 20–89 × 3–7 μm, smooth and thin-walled; terminal cells cylindrical, apically narrow, 24–61 μm in length, the apex 3–4 μm and base 4–7 μm in diam., thin-walled, hyaline. ***Hypodermium*** formed by fusiform to subglobose hyphae, 21–41 × 17–25 μm, thin-walled, hyaline. ***Lamellar trama*** subregular, dextrinoid. ***Stipitipellis*** a cutis composed of hyphae 3–8 μm in diam., smooth, thin-walled, with projecting hyphae 3–8 μm in diam.; ***caulocystidia*** 29–47 × 6–12 μm, clavate and apices tapered, thick-walled (0.5–0.6 μm), smooth. ***Clamps*** present in all tissues.

##### Habitat.

Scattered on the litter layer in *Pinuskoraiensis*, *Larixgmelinii* and *Tilia* sp. mixed forests.

##### Known distribution.

Heilongjiang Province, China.

##### Additional material examined.

China. Heilongjiang Province: Liangshui National Nature Reserve, Yichun City, 21 August 2021, Zewei Liu, Yupeng Ge, Qin Na and Shixin Wang, *FFAAS0419* (collection number MY0654); same location, 21 August 2021, Zewei Liu, Yupeng Ge, Qin Na and Shixin Wang, *FFAAS0420* (collection number MY0657); Heilongjiang Province: Taipinggou National Nature Reserve, Hegang City, 3 September 2021, Shixin Wang, *FFAAS0426* (collection number MY0795).

##### Notes.

Cheilocystidia with a long and narrow protuberance is the key microscopic character that distinguishes *M.subulata* and is uncommon in sect. Calodontes (Smith 1947; Maas Geesteranus 1992a, 1992b; Grgurinovic 2003; Robich 2003; Chew et al. 2014; Aravindakshan and Manimohan 2015; Aronsen and Læssøe 2016; Na 2019; Liu et al. 2021). *Mycenalammiensis* Harmaja and *M.pelianthina* (Fr.) Quél. have similar cheilocystidia, but differ from *M.subulata* by their broader cheilocystidia with purplish-brown contents and having pleurocystidia (Smith 1947; Robich 2003; Aronsen and Læssøe 2016). The cheilocystidia of *M.subcorticalis* (Cooke & Massee) Sacc. with a protuberance are similar to those of *M.subulata*. However, *M.subcorticalis* has larger and inamyloid spores, a gelatinised pileipellis and a stipitipellis with sparse excrescences (Grgurinovic 2003). More rarely, mucronate cheilocystidia and absence of pleurocystidia have been described for *M.pearsoniana* and its clay pink pileus is similar to that of *M.subulata*, but *M.pearsoniana* differs in having a slightly glutinous pileus when wet and inamyloid spores (Aronsen and Læssøe 2016; Na 2019). Other species that are macroscopically similar to *M.subulata*, namely *M.luteovariegata*, *M.nullawarrensis* and *M.pura*, can be distinguished by cheilocystidia shape and presence of pleurocystidia (Perry 2002; Robich 2003; Grgurinovic 2003; Harder et al. 2013; Aronsen and Læssøe 2016; Na 2019).

**Figure 13.**
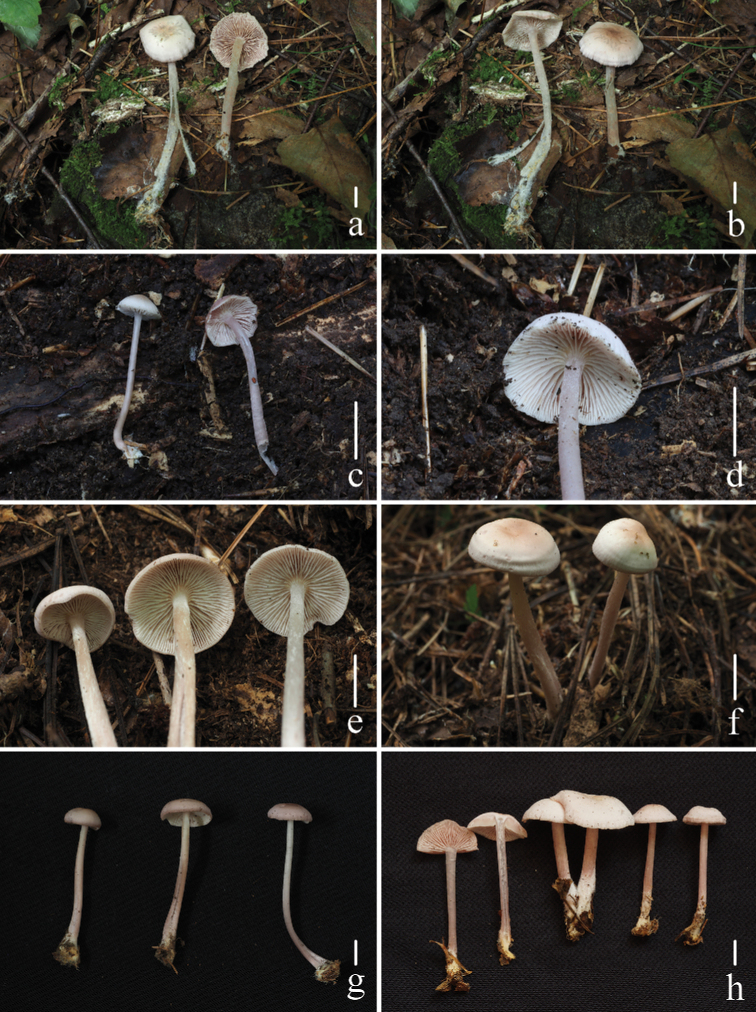
Basidiomata of *Mycenasubulata* Z.W. Liu, Y.P. Ge & Q. Na **a, b***FFAAS0419***c, d***FFAAS0420***e–g***FFAAS0423*, holotype **h***FFAAS0426* Scale bars: 10 mm (**a–h**). Photographs **a, b, e, f** by Yupeng Ge **c, d** by Qin Na **g** by Zewei Liu **h** by Shixin Wang.

**Figure 14.**
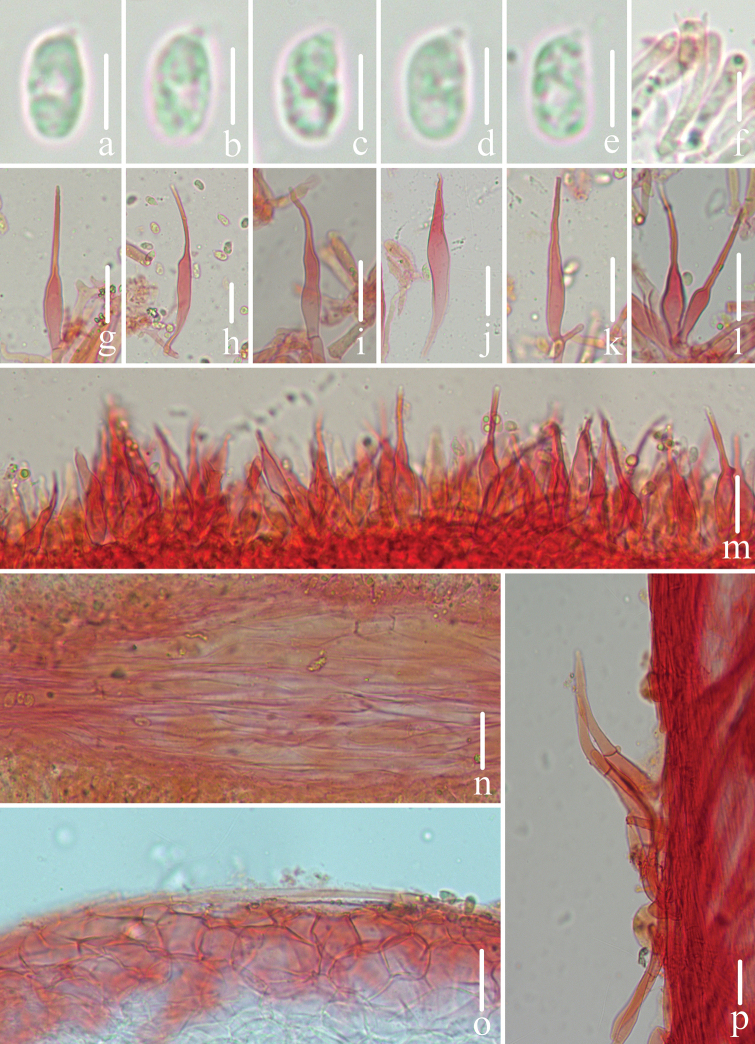
Microscopic features of *Mycenasubulata* (*FFAAS0423*, holotype) **a–e** basidiospores **f** basidia **g–m** cheilocystidia **n** lamellar trama **o** pileipellis and hypodermium **p** stipitipellis and caulocystidia. Scale bars: 5 μm (**a–e**); 10 μm (**f**); 20 μm (**g–p**).

**Figure 15.**
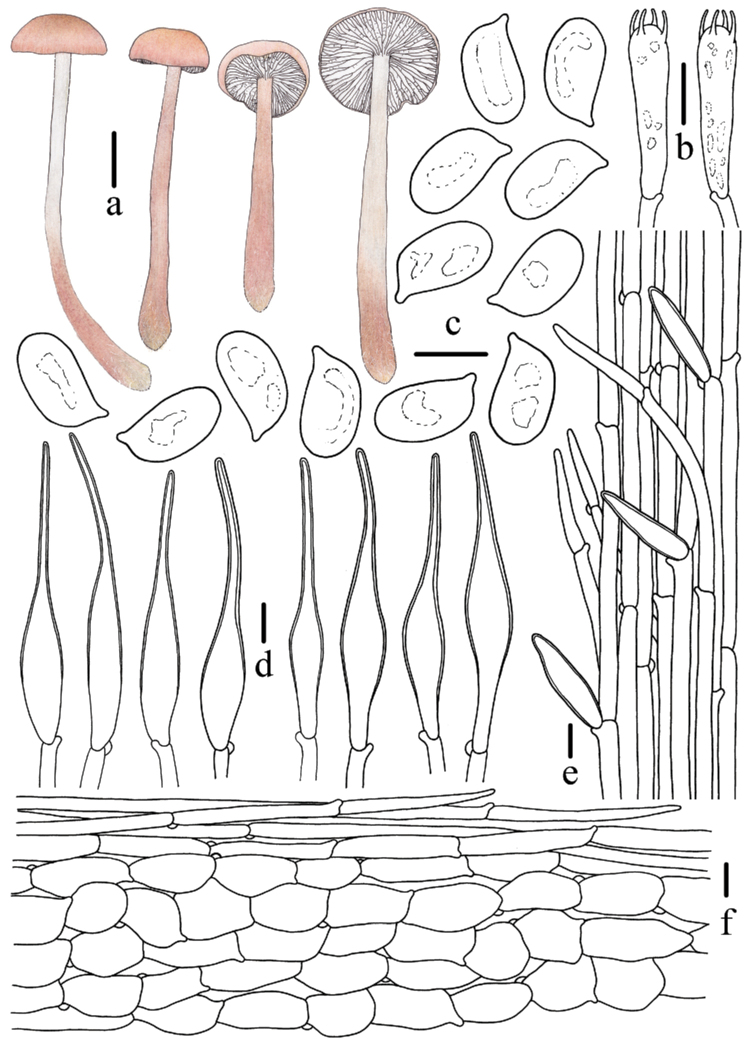
Morphological features of *Mycenasubulata* (*FFAAS0423*, holotype) **a** basidiomata **b** basidia **c** basidiospores **d** cheilocystidia **e** stipitipellis and caulocystidia **f** pileipellis and hypodermium. Scale bars: 10 mm (**a**); 5 μm (**c**); 10 μm (**b, d–f**). Drawings by Zewei Liu.

### ﻿Key to species of sect. Calodontes known worldwide

**Table:** 

1	Stipe white	**2**
–	Stipe coloured	**4**
2	Pileus white	***Mycenasubaquosa***
–	Pileus coloure	**3**
3	Pileus pink and lamellae emarginate, pileipellis without inflated terminal cells	***Mycenarosea***
–	Pileus brown and lamellae adnate, pileipellis with fusiform, subcylindrical to lageniform terminal cells	***Mycenadura***
4	Lamellae edge with coloured dots	**5**
–	Lamellae edge white or without dots	**6**
5	Caulocystidia present, spores almost broader than 4 μm	***Mycenalammiensis***
–	Caulocystidia absent, spores almost narrower than 4 μm	***Mycenapelianthina***
6	Basidiospores inamyloid	**7**
–	Basidiospores amyloid	**10**
7	Pleurocystidia absent	**8**
–	Pleurocystidia present	**9**
8	Stipitipellis and caulocystidia smooth	***Mycenapearsoniana***
–	Stipitipellis and caulocystidia with nodulose excrescences	***Mycenasubcorticalis***
9	Pileipellis gelatinised, caulocystidia absent, cheilo- and pleurocytsidia base uncontracted, disc greyish-red or orange white in pileus	***Mycenasirayuktha***
–	Pileipellis not gelatinised, caulocystidia present, cheilo- and pleurocytsidia base contracted, disc wood brown or reddish-brown in pileus	***Mycenavinacea***
10	Pleurocystidia present	**11**
–	Pleurocystidia absent	**16**
11	Caulocystidia absent, almost cheilocystidia apically mucronate or subcapitate	***Mycenacahaya***
–	Caulocystidia present, almost cheilocystidia apically broadly rounded	**12**
12	Caulocystidia with apical excrescences, spores more than 5.6 μm width	***Mycenaclarkeana***
–	Caulocystidia without apical excrescences, spores less than 5.6 μm width	**13**
13	Cheilocystidia base uncontracted	***Mycenapolycystidiata***
–	Cheilocystidia base contracted	**14**
14	Stipe brown to dark brown, Q_av_ = 1.5	***Mycenanullawarrensis***
–	Stipe not brown to dark brown, Q_av_ > 1.5	**15**
15	Pileus sulphur yellow to reddish-grey, stipe reddish-grey	***Mycenaluteovariegata***
–	Pileus generally pinkish or purplish, stipe whitish to pinkish-purple	***Mycenapura***
16	Caulocystidia absent	**17**
–	Caulocystidia present	**18**
17	Pileus brown to dark brown, spores weakly amyloid	***Mycenaseminau***
–	Pileus brownish-orange to greyish-yellow, spores amyloid	***Mycenasinar***
18	Cheilocystidia slender fusiform, with distinctly long and narrow protuberance	***Mycenasubulata***
–	Cheilocystidia clavate, utriform, subfusiform, or subcylindrical, with short mucronate or none	**19**
19	Spores less than 6 μm length	***Mycenakuehneriana***
–	Spores more than 6 μm length	**20**
20	Pileus more than 35 mm in diam., lamellae dark brownish-violet to reddish-violet	***Mycenadiosma***
–	Pileus less than 35 mm in diam., lamellae white	**21**
21	Lamellar trama subcellular, pileus lilac to purple	***Mycenayuezhuoi***
–	Lamellar trama subregular, pileus brownish	**22**
22	Cheilocystidia utriform, sometimes clavate, thin-walled, lamellae adnexed to emarginate	***Mycenarufobrunnea***
–	Cheilocystidia clavate with slightly inflated apex, thick-walled, lamellae sinuate to subdecurrent	***Mycenashengshanensis***

## ﻿Discussion

Maas Geesteranus (1980) proposed that Mycenasect.Calodontes could be divided into three subsections based on the colour of the lamellar edge and the amyloid reaction of the basidiospores. Subsequently, taxonomists have followed this division, but opinions have differed on the diagnostic characters that support this classification (Grgurinovic 2003; Robich 2003; Harder et al. 2010; Chew et al. 2014). Some taxonomists classified the subsections according to the amyloid reaction of basidiospores, cheilocystidia and pleurocystidia contents and presence or absence of pleurocystidia, but the shapes of cheilocystidia and pleurocystidia were not considered (Grgurinovic 2003; Harder et al. 2010; Chew et al. 2014). Robich (2003) also did not consider the shapes of cheilocystidia and pleurocystidia, but the colour of the lamellar edge and cheilocystidia contents were emphasised to distinguish subsections. According to the historical infrasectional classification of sect. Calodontes, *M.polycystidiata* could be classified in subsect. Purae, whereas *M.rufobrunnea*, *M.shengshanensis* and *M.subulata* cannot be assigned to a subsection owing to their having amyloid spores and lacking pleurocystidia (Maas Geesteranus 1980; Harder et al. 2010).

Phylogenetic reconstructions do not fully support recognition of three subsections defined by morphological characters; notably, subsect. Violacellae and subsect. Purae are polyphyletic in the phylogenies (Harder et al. 2010, 2012, 2013). Chew et al. (2014) supported the views of Harder et al. (2010, 2012) and the new taxa proposed by the former authors were not assigned to a subsection. Additionally, subsect. Purae was proved to be polyphyletic in our combined analysis of ITS, *rpb1* and *tef1* dataset, which also supported analysis, based on single gene region (Harder et al. 2013).

The five taxa of Mycenasect.Calodontes recorded from China show obvious differences in pileus colour and in the shapes of cheilocystidia and pleurocystidia (if present) (Liu et al. 2021). The colour of the pileus includes greyish-rose, reddish-grey, purple, reddish-brown and violet-brown and most show a gradual transition with age. Clavate, obclavate, utriform and fusiform cheilocystidia with a long, narrow protuberance are observed, but pleurocystidia are present only in *M.polycystidiata*. Forms and variations within *M.pura* complex had a wide range of pileus colour, but the shape of cheilocystidia was highly similar and could be clearly distinguished from the four new taxa (Robich 2003).

In our phylogenetic analysis, four new species all formed separate clades with high support and had obvious genetic distance from other species in sect. Calodontes. *Mycenarufobrunnea* is more closely related to the phylogenetic species within *M.pura* complex by Harder et al. (2013). While the other three new species are significantly more distant from *M.pura* complex genetically, *M.shengshanensis* and *M.subulata*, formed a sister relationship with high support from *M.pearsoniana*; *M.polycystidiata* clustered with *M.diosma*, but is poorly supported.

Based on extensive field work in China, most specimens of sect. Calodontes have been observed in coniferous forests or mixed coniferous-broadleaved forests in early autumn (Na 2019; Liu et al. 2021). Specimens of the four new taxa described in the present study were collected from Changbai Mountain and the Lesser Khinggan Mountains in northeast China from mixed broadleaf-Korean pine (*Pinuskoraiensis*) forests (Zhao et al. 2004; Wang and Guo 2016). In particular, *M.polycystidiata* and *M.subulata* were both distributed in the Liangshui National Nature Reserve on the Lesser Khinggan Mountains, where the dominant forest species is *P.koraiensis*, mixed with fewer *Betula*, *Tilia*, *Quercus* and *Picea* individuals (She et al. 2022). Moreover, more specimens were located in the northern region of China with an average temperature not more than 20 °C in August. For example, the average temperature is 16.4 °C in Liangshui National Nature Reserve and 16.3 °C in Shengshan National Nature Reserve (Liu 2017). Therefore, we speculate that members of this section in China prefer the climate types Dwa, Dwb and Dwc according to the Köppen climate classification (Kottek et al. 2006; Wang et al. 2020).

## Supplementary Material

XML Treatment for Mycena polycystidiata

XML Treatment for Mycena rufobrunnea

XML Treatment for Mycena shengshanensis

XML Treatment for Mycena subulata
